# Whirlin, a cytoskeletal scaffolding protein, stabilizes the paranodal region and axonal cytoskeleton in myelinated axons

**DOI:** 10.1186/1471-2202-14-96

**Published:** 2013-09-06

**Authors:** James A Green, Jun Yang, M’hamed Grati, Bechara Kachar, Manzoor A Bhat

**Affiliations:** 1 Department of Cell and Molecular Physiology, University of North Carolina School of Medicine, Chapel Hill, NC 27599, USA; 2 Ophthalmology and Visual Sciences, Moran Eye Center and Neurobiology and Anatomy, University of Utah, Salt Lake City, UT 84132, USA; 3 Laboratory of Cell Structure and Dynamics, National Institute on Deafness and Other Communication Disorders, National Institutes of Health, Bethesda, MD 20892, USA; 4 Department of Physiology, University of Texas School of Medicine, Health Science Center, 7703 Floyd Curl Drive, San Antonio, TX 78229, USA

**Keywords:** Myelinated axons, Axonal domains, Paranodal domain, Axonal cytoskeleton, Whirlin

## Abstract

**Background:**

Myelinated axons are organized into distinct subcellular and molecular regions. Without proper organization, electrical nerve conduction is delayed, resulting in detrimental physiological outcomes. One such region is the paranode where axo-glial septate junctions act as a molecular fence to separate the sodium (Na^+^) channel-enriched node from the potassium (K^+^) channel-enriched juxtaparanode. A significant lack of knowledge remains as to cytoskeletal proteins which stabilize paranodal domains and underlying cytoskeleton. Whirlin (Whrn) is a PDZ domain-containing cytoskeletal scaffold whose absence in humans results in Usher Syndromes or variable deafness-blindness syndromes. Mutant *Whirlin* (*Whrn*) mouse model studies have linked such behavioral deficits to improper localization of critical transmembrane protein complexes in the ear and eye. Until now, no reports exist about the function of Whrn in myelinated axons.

**Results:**

RT-PCR and immunoblot analyses revealed expression of *Whrn* mRNA and Whrn full-length protein, respectively, in several stages of central and peripheral nervous system development. Comparing wild-type mice to *Whrn* knockout (*Whrn*^*−/−*^) mice, we observed no significant differences in the expression of standard axonal domain markers by immunoblot analysis but observed and quantified a novel paranodal compaction phenotype in 4 to 8 week-old *Whrn*^*−/−*^ nerves. The paranodal compaction phenotype and associated cytoskeletal disruption was observed in *Whrn*^*−/−*^ mutant sciatic nerves and spinal cord fibers from early (2 week-old) to late (1 year-old) stages of development. Light and electron microscopic analyses of *Whrn* knockout mice reveal bead-like swellings in cerebellar Purkinje axons containing mitochondria and vesicles by both. These data suggest that Whrn plays a role in proper cytoskeletal organization in myelinated axons.

**Conclusions:**

Domain organization in myelinated axons remains a complex developmental process. Here we demonstrate that loss of Whrn disrupts proper axonal domain organization. Whrn likely contributes to the stabilization of paranodal myelin loops and axonal cytoskeleton through yet unconfirmed cytoskeletal proteins. Paranodal abnormalities are consistently observed throughout development (2 wk-1 yr) and similar between central and peripheral nervous systems. In conclusion, our observations suggest that Whrn is not required for the organization of axonal domains, but once organized, Whrn acts as a cytoskeletal linker to ensure proper paranodal compaction and stabilization of the axonal cytoskeleton in myelinated axons.

## Background

Nervous system function depends on proper molecular organization between neurons and glial cells. In myelinated neurons, the segregation and enrichment of proteins in the defined domains, the Node of Ranvier, paranode, and juxtaparanode, is critical for saltatory action potential propagation [1-4]. Without proper organization, electrical nerve conduction is delayed and can result in significant motor and sensory deficits. The paranodal domain is a region of direct interaction between the glial myelin membrane loops and neuronal plasma membrane. Axo-glial septate junctions (AGSJ) link these glial membrane loops to the axonal membrane and establish the paranodal ionic barrier separating sodium (Na^+^) channel-enriched node from the potassium (K^+^) channel-enriched juxtaparanode [5]. AGSJ’s are composed of three transmembrane proteins: Contactin (Cont) [6], Contactin-associated protein (Caspr) [7], and Neurofascin155 (glial-derived 155 kDa isoform) [8]. Genetic ablation of any of the three molecules disrupts the paranodal barrier function and results in degraded action potential propagation [6-11]. While much research has focused on these three proteins, other molecules contribute to domain formation and stabilization. For example, Caspr2 and TAG-1 are required for the organization of the juxtaparanodal domain and localization of potassium (K^+^) channels [12]. For long-term stability, Caspr and Caspr2 are thought to rely on cytoskeletal scaffold proteins to link their associated complexes with the axonal cytoskeleton. Loss of Caspr results in significant cytoskeletal disorder [9], and since its extracellular partner Cont lacks an intracellular c-terminus, the c-terminus of Caspr is likely a critical region of interaction between AGSJ’s and the axonal cytoskeleton. Previous studies using *4.1B* mutant mice revealed that the 4.1B cytoskeletal protein contributes to paranodal and juxtaparanodal stability [13,14]. A recent report also highlights 4.1G as an organizer of internodes in the peripheral nervous system [15]. The neuron is abundant with cytoskeletal scaffolds however, and such findings do not exclude the possibility that other cytoskeletal elements may contribute to paranodal maintenance and long-term stability.

Whrn is a cytoskeletal scaffold protein that plays an important role in vision and hearing [16,17]. In humans, *WHIRLIN* (*WHRN*) (*DFNB31*) mutations have been linked to Usher syndrome Type II (USH2), an autosomal recessive vision-hearing impairment disorder [18,19]. In mice, the *Whrn* coding sequence consists of 12 exons with two dominant splice variants, a full length and short (exon 5–12) isoform. Both variants contain a Proline-rich domain and PDZ-domain(s) which have been shown to link submembranous cytoskeletal elements to transmembrane complexes, as well as to self-oligomerize [20]. In the eye, Whrn interacts with the transmembrane proteins Usherin (USH2) and Very Large G protein-coupled Receptor-1 (VLGR1/GPR98) within the periciliary membrane complex of photoreceptors. Likewise in the inner ear, Whrn interacts with Usherin and VLGR1/GPR98, which form the stereociliary ankle-links. Additionally, Whrn interacts indirectly with 4.1B [21] and 4.1R through Mpp1/p55 [22], and directly with Myosin XVa [23]. Finally, Whrn is implicated with Esp8 in stereocilia-length regulation [24]. While the function of Whrn in the ear and the eye has received significant attention, very little is known about its function in the central and/or peripheral nervous system. There are reports of Whrn protein expression in the cerebrum, cerebellum, and brainstem in wild-type mice and the protein is absent in *Whrn* knockout (*Whrn*^*−/−*^) and *whirler* (*Whrn*^*wi/wi*^) mutant mice [25]. In *Drosophila*, the closest homolog to *Whrn* is *dyschronic* (*dysc*) [26]. In *dysc* mutants there is arrhythmic locomotor behavior but the eclosion circadian rhythms and clock protein cycling is unaffected [26]. Here we report that Whrn is involved in proper compaction of the paranodal region in myelinated axons and for proper stabilization of the axonal cytoskeleton.

## Results and discussion

### Whrn is expressed in central and peripheral nervous system tissues throughout development

**Figure 1:**
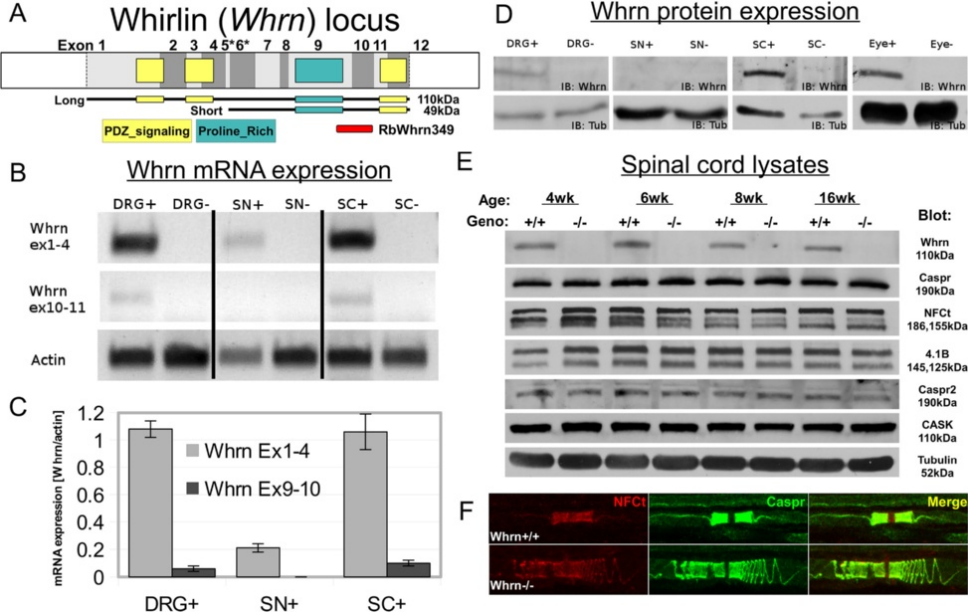
**Whirlin (Whrn) is a PDZ-containing protein expressed throughout the central and peripheral nervous system. A**. Schematic showing the relative organization of the twelve exons which make up the *Whrn* full-length sequence including untranslated exon regions (white boxes) and coding sequence (alternating grey boxes). *Whrn’s* second, short isoform begins with an alternative transcriptional start site (asterisks). Both variants contain PDZ-domains (yellow boxes) and a Proline-rich domain (blue box). A red rectangle highlights the region used for antibody creation (RbWhrn349). **B**. RT-PCR analysis shows absence of any *Whrn* transcripts in homozygous *Whrn* exon 1 knockout mice from dorsal root ganglia (DRG, peripheral neuronal nuclei), sciatic nerves (SN, peripheral glial nuclei), and spinal cords (SC, combination neuronal/glial nuclei). mRNA transcripts were reverse transcribed and amplified using primers located on *Whrn* exon 1 and 4 (top panel), *Whrn* exon 9 and 10 (middle panel), or actin (bottom panel). **C**. Relative quantification of *Whrn* mRNA from Figure 1B expressed as a ratio of *Whrn* to actin band intensity. **D**. Immunoblots of 110 kDa Whrn protein band in wild-type and *Whrn* mutant DRG, SN, SC, and whole eye using affinity-purified RbWhrn349 antibody. αTubulin served as a loading control. **E**. Immunoblots of wild-type and *Whrn* knockout mutant 4, 6, 8, and 16-week-old spinal cord lysates. The expression profile using various myelinated axonal domain markers includes Caspr, Neurofascin (186 and 155), 4.1B, Caspr2, as well as CASK. αTubulin served as a loading control. **F**. Immunofluorescence of teased sciatic nerve fibers from wild-type (upper panel) and *Whrn* knockout mice (lower panel) mice. Neurofascin (NFCt, red) and paranodal Caspr (green) reveal paranodal compaction is disrupted in *Whrn* knockout fibers. Note NFCt (red) detects both paranodal NF155 and nodal NF186 isoforms.

Whrn is a cytoskeletal scaffolding protein which functions to link membrane protein complexes to the cytoskeleton within hair cell stereocilia of the ear and photoreceptors in the eye. To begin assessing its function in myelinated neurons, we obtained *Whrn* exon 1 knockout mice [17]. As reported previously, the murine *Whrn* locus consists of 12 exons with two dominant splice variants, a full length (~4 kb) isoform and a short (~2.5 kb) isoform (Figure 1A). Both variants contain PDZ-domains (Figure 1A, yellow box) and a Proline-rich domain (Figure 1A, blue box). After initial back crossing to *C57BL6* mouse strain, we identified and confirmed the *Whrn* genotype using PCR methods. To begin characterizing mRNA expression of *Whrn* in the central (CNS) and peripheral nervous systems (PNS), we examined the relative expression of *Whrn* by reverse transcriptase polymerase chain reaction (RT-PCR) in dorsal root ganglia (DRG), sciatic nerves (SN), and spinal cord (SC) tissues (Figure 1B) in postnatal 21 day-old mice. With this subset of tissues we could delineate the origin of *Whrn* expression as SC tissue is a combination of glial and neuronal nuclei, DRG is predominantly neuronal, and SN is principally glial cytoplasm and nuclei. Since Whrn has two major isoforms, we designed specific primers to distinguish the full length isoform (Exons 1–4) alone and those common to the full length and short isoforms (Exons 9–10). No expression of Whrn isoforms was observed in *Whrn*^*−/−*^ mice (Figure 1B). Robust expression of *Whrn* (Exons 1–4) mRNA was observed in DRG and SC tissue while weak expression was observed in SN tissue. Interestingly, weak expression of *Whrn* (Exons 9–10) mRNA was observed in DRG and SC tissue but no significant expression was observed in SN tissue. *Actin* (Exons 2–4) mRNA expression was used as a control for total RNA present. Relative expression was quantified as a ratio of *Whrn* to *Actin* mRNA between three total RT-PCR analyses (Figure 1C). After finding *Whrn* mRNA expression in the wild-type CNS and PNS neuronal tissues, we next sought to determine its protein expression in P30 *Whrn*^*+/+*^ versus *Whrn*^*−/−*^ mice by immunoblot analysis. In order to pursue these experiments, we generated several antibodies and affinity-purified one to the non-domain encoding, c-terminal region (aa699-804) of Whrn (Figure 1A, red bar). After affinity-purification, wild-type (+) and *Whrn* knockout (−) lysates derived from DRG, SN, SC, and whole eye were immunoblotted (Figure 1D, upper blots). Whole eye lysates were used as a positive control for protein expression based on previous reports [17]. A 110 kDa Whrn band representing the full length protein was present in DRG, SC, and whole eye lysates. No 110 kDa Whrn band was observed in *Whrn*^*−/−*^ lysates or in wild-type SN lysate. Each tissue type showed similar total protein levels between genotypes based on total Tubulin on immunoblots (Figure 1D, lower blots). Having confirmed *Whrn*^*−/−*^ tissues were deficient in *Whrn* mRNA and protein, we next sought to determine if loss of Whrn resulted in altered protein expression of known nodal, paranodal, and juxtaparanodal proteins. We prepared spinal cord lysates from 4, 6, 8, and 16 week-old wild-type (+/+) and *Whrn* knockout (−/−) mice and immunoblotted for Whrn (110 kDa) (Figure 1E). Whrn protein expression levels were similar from 4 to 16 weeks in wild-type mice while no expression was observed in the *Whrn*^*−/−*^ at any time point. Next, we immunoblotted for the paranodal protein Caspr (190 kDa) and found similar expression levels between 4–16 week-old in both *Whrn*^*+/+*^ and *Whrn*^*−/−*^ mice. Using a pan-Neurofascin-c-terminal (NFct) antibody, we observed similar levels of nodal Neurofascin186 and paranodal Neurofascin155 protein expression in wild-type and *Whrn* knockout mice across 4–16 weeks of age. Examination of neuronal cytoskeletal protein 4.1B and juxtaparanodal protein Caspr2 shows steady protein expression levels in wild-type and *Whrn* knockout mice from 4–16 weeks of age. Finally, previous reports showed *in vivo* interaction between rat Whrn/CIP98 and calmodulin-dependent serine kinase (CASK) [27], a synaptic organization protein. After immunoblotting for CASK, no difference in its protein level was observed across *Whrn* genotype or ages from 4–16 weeks. Total protein levels in spinal cord lysates were consistent across the ages and genotypes based on Tubulin levels (Figure 1E). In summary, these developmental expression profiles suggest that loss of Whrn expression does not affect the overall expression or stability of other axonal domain markers.

### *Whrn* knockout mice reveal a quantifiable paranodal compaction phenotype in peripheral myelinated axons

To determine the effects of Whrn loss on axonal domain organization, we examined well-characterized nodal, paranodal, and juxtaparanodal markers by immunofluorescence in wild-type and *Whrn*^*−/−*^ mice. Extensive and repeated immunostaining with our Whrn antibody revealed no consistent localization or differences in Whrn localization between wild-type to *Whrn*^*−/−*^ fibers (data not shown). Compared to wild-type, the most striking observation in teased *Whrn*^*−/−*^ sciatic nerve fibers was the spring-like separation (Figure 1F) of the paranodal axo-glial septate junction (AGSJ) loops beginning along the paranodal-juxtaparanodal border as observed by Caspr and NF155 immunostaining. Irregular paranodal compaction is rarely observed in wild-type fibers, so we sought to quantify the overall observation of this phenotype in *Whrn*^*+/+*^ and *Whrn*^*−/−*^ fibers. We utilized a blinded counting strategy to count spring-like phenotypes from wild-type (N = 4676) or *Whrn*^*−/−*^ (N = 2798) Caspr-stained paranodes. A statistically significant difference (student t-test p = 0.02) was observed in the percentage of irregular paranodal compaction at 0.3% (SEM=0.07%) in wild-type fibers compared to 1.8% (SEM=0.45%) in *Whrn* knockout mice. Having confirmed the significance of this spring-like paranodal phenotype in 7 week-old *Whrn* knockout fibers, we expanded our analysis to 4–8 week-old time points.

**Figure 2:**
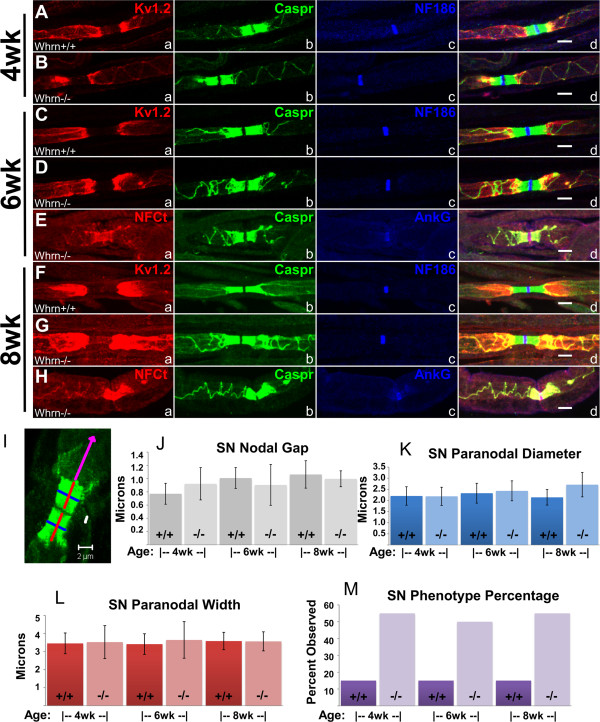
**Loss of Whirlin in the peripheral nervous system results in disrupted paranodal compaction. A**–**H**. 4, 6, 8-week-old teased sciatic nerve fibers either wild-type **(Aa**–**Ad**, **Ca**–**Cd**, **Fa**–**Fd)** or Whrn knockout **(Ba**–**Bd**, **Da**–**Dd**, **Ea**–**Ed**, **Ga**–**Gd**, **Ha**–**Hd)** immunostained against K_v_1.2 **(Aa**-**Da**, **Fa**, **Ga**, red**)**, NFCt **(Ea**, **Ha**, red**)**, Caspr **(Ab**–**Hb**, green**)**, NF186 **(Ac**-**Dc**, **Fc**, **Gc**, blue**)**, AnkG **(Ec**, **Hc**, blue**)**, and merged images **(Ad**–**Hd)**. In all *Whrn* mutant panels, Caspr **(Bb**, **Bd**; **Db**, **Dd**; **Eb**, **Ed**; **Gb**, **Gd**; **Hb**, **Hd,** green**)** and paranodal NF155 (NFCt) **(Ea**, **Ed**; **Ha**, **Hd**, red**)** fail to compact properly at the paranodes. Nodal NF186 or AnkG are not affected **(Ac**,**d**–**Hc**,**d**, blue**)**. Scale bars **(Ad**-**Hd)** = 5 μm. **I**. Sample image shows parameters of various domain measurements in (nodal gap in white, paranodal diameter in blue, paranodal width in red, and counting of spring-like phenotype in purple) using ~10 micron caliber, Caspr-immunostained wild-type and *Whrn*^*−/−*^ fibers. **J**-**L**. No statistically significant differences were observed comparing 4, 6, and 8-week-old wild-type and mutant fibers with concern to nodal gap **(J)**, paranodal diameter **(K)**, or paranodal width **(L)** (N=20 for each genotype/age combination). Note the greater percentage of paranodes with compaction issues in mutant fibers **(M**, light purple bars**)** likely contributes to the increased deviation in paranodal widths **(L**, light red bars**)**.

Sciatic nerves from *Whrn*^*+/+*^ or *Whrn*^*−/−*^ littermate mice at 4, 6, and 8 weeks, were immunostained with antibodies against nodal, paranodal, and juxtaparanodal markers (Figure 2). In the 4 week-old wild-type sciatic nerve (Figure 2Aa-d), juxtaparanodal K_v_1.2 (Figure 2Aa) was separated from nodal NF186 (Figure 2Ac) by paranodal Caspr (Figure 2Ab). This demarcation of domains (Figure 2Ad) in myelinated fibers is also observed in 4 week-old *Whrn*^*−/−*^ fibers (Figure 2Bd). Myelin loops of AGSJs in 4 week-old *Whrn*^*−/−*^ sciatic nerve fibers (Figure 2Bb) were already separating away from the paranode compared to 4 week-old wild-type sciatic nerve fibers (Figure 2Ab). Breaks in K_v_1.2 localization (Figure 2Ba) were observed even where Caspr staining (Figure 2Bb) was limited to a single myelin loop, suggesting AGSJ’s barrier function was conserved. Juxtaparanodal localization was diffuse and asymmetric in *Whrn*^*−/−*^ fibers compared to wild-type (Figure 2Aa vs. 2Ba). When comparing wild-type to *Whrn* knockout mice, we observed no obvious differences in intensity levels of juxtaparanodal (Figure 2Aa vs. 2Ba) or nodal (Figure 2Ac vs. 2Bc) markers. At 6 weeks, teased wild-type sciatic nerves (Figure 2Ca-d) showed typical domain organization. 6 week-old *Whrn* null sciatic nerves have typical NF186 (Figure 2Dc) nodal domain organization while Caspr paranodal compaction (Figure 2Db) and K_v_1.2 juxtaparanodal (Figure 2Da) localization were disrupted. Additional immunostaining in 6 week-old *Whrn* null sciatic nerves with a pan-Neurofascin-C-terminal (NFct) antibody (Figure 2Ea) detected both nodal NF186 and paranodal NF155. Paranodal NF155 (Figure 2Ea, non-nodal signal) and Caspr (Figure 2Eb) immunostaining mostly colocalize (Figure 2Ed) in *Whrn*^*−/−*^ sciatic nerve fibers and revealed similar problems with paranodal compaction. AnkG nodal (Figure 2Ec) domain organization in 6 week-old *Whrn*^*−/−*^ sciatic nerve fibers appeared similar to wild-type (Figure 2Cc). Finally, immunostained 8 week-old sciatic nerves revealed similar NF186 nodal (Figure 2Fc vs. 2Gc) and AnkG nodal organization (Figure 2Hc). Like 4 and 6 week-old sciatic nerves, Caspr (Figure 2Gb, Hb) and NFct (Figure 2Ha) immunostaining consistently revealed paranodal compaction defects as well as K_v_1.2 juxtaparanodal (Figure 2Fa vs. 2Ga) diffusion in *Whrn* knockout mice when compared with wild-type (Figure 2Fb). To determine if such peripheral nerve phenotypes could be the result of differences in inner mesaxons, we immunostaining 7 week-old wild-type and *Whrn* knockout fibers with MAG but observed no striking phenotypic differences in localization (data not shown). In summary, the paranodal and juxtaparanodal regions displayed phenotypes that suggest that normal compaction of the paranodal loops fails to occur in *Whrn* knockout mice at 4, 6, and 8 weeks of age.

During initial quantification we observed that larger diameter myelinated fibers had proportionally more paranode compaction defects compared to thinner caliber myelinated fibers, so we imaged and assembled twenty Caspr-stained confocal images for each genotype and time point (4, 6, and 8 week-old) to assess any subtle, sub-micron paranodal changes by light microscopy due to *Whrn* loss. Next, we measured various dimensions of the paranode (Figure 2I). Note images collected were from ~10 micron caliber myelinated neurons. Using Zeiss software, micron measurements were recorded, tabulated, and reported as averages with standard deviations for the nodal gap (Figure 2J), the paranodal diameter (Figure 2K), paranodal width (Figure 2L), as well as the percentage of paranodes which display paranodal compaction abnormalities (Figure 2M). The nodal gap (Figure 2J) for wild-type and *Whrn*^*−/−*^ sciatic nerves was 0.77±0.16 μm and 0.92±0.24 μm at 4 weeks, 1.01±0.16 μm and 0.91±0.31 μm at 6 weeks, and 1.06±0.21 μm and 1.00±0.12 at 8 weeks respectively. No statistically significant difference was noted between ages or genotypes with respect to nodal gap. The paranodal diameter (Figure 2K) for wild-type and *Whrn*^*−/−*^ sciatic nerves was 2.20±0.42 μm and 2.19±0.41 μm at 4 weeks, 2.33±0.45 μm and 2.43±0.45 μm at 6 weeks, and 2.14±0.35 μm and 2.71±0.55 at 8 weeks respectively. With respect to paranodal diameter, measurements were similar between all groups. The paranodal width (Figure 2L) for wild-type and *Whrn*^*−/−*^ sciatic nerves was 3.45±0.58 μm and 3.52±0.91 μm at 4 weeks, 3.41±0.57 μm and 3.64±1.01 μm at 6 weeks, and 3.58±0.48 μm and 3.56±0.53 at 8 weeks respectively. No statistically significant difference was found in paranodal width. The paranodal compaction phenotype percentage (Figure 2M) for wild-type and *Whrn*^*−/−*^ sciatic nerves was 15% (3/20) and 55% (11/20) at 4 weeks, 15% (3/20) and 50% (10/20) at 6 weeks, and 15% (3/20) and 55% (11/20) respectively. As a reminder, the increase in the Whrn knockout phenotype percentage represents the shift in selection from all fibers (1.8%) to larger-caliber myelinated fibers (50%). Note the greater percentage of paranodes with compaction issues in *Whrn*^*−/−*^ sciatic nerves (Figure 2M, light purple bars) likely contributes to the increased deviation in paranodal widths (Figure 2L, light red bars). Additionally, comparisons between 8 week-old wild-type and *Whrn*^*−/−*^ sciatic nerve fibers demonstrated no significant difference in the conduction velocity (average ~30 m/s) or waveforms (data not shown) of measured compound action potentials in two separate measurement trials.

### *Whrn* knockout mouse sciatic nerve and spinal cord myelinated fibers display paranodal compaction abnormalities throughout development

**Figure 3:**
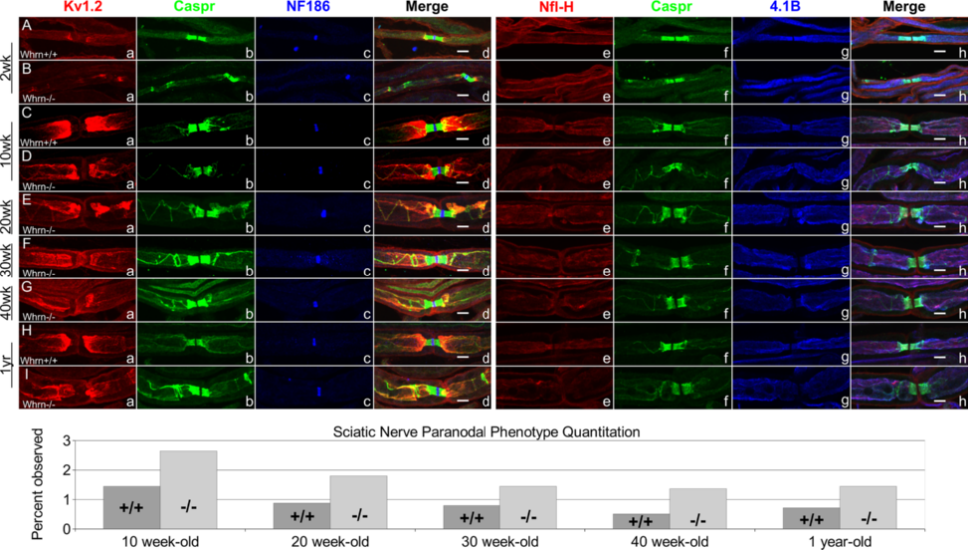
**Loss of Whirlin contributes to abnormal paranodal compaction and cytoskeleton instability throughout sciatic nerve age. A**–**I**. 2-week, 10, 20, 30, 40-week, and 1-yr-old teased sciatic nerve fibers either wild-type **(Aa**–**Ah**, **Ca**–**Ch**, **Ha**–**Hh)** or *Whrn* knockout **(Ba**–**Bh**, **Da**–**Dh**, **Ea**–**Eh**, **Fa**–**Fh**, **Ga**–**Gh**, **Ia**–**Ih)** immunostained against K_v_1.2 **(Aa**-**Ia**, red**)**, Caspr **(Ab**,**f**–**Ib**,**f**, green**)**, NF186 **(Ac**-**Ic**, blue**)**, heavy chain Neurofilament/Nfl-H **(Ae**-**Ie**, red**)**, Protein 4.1 band/4.1B **(Ag**-**Ig**, blue**)**, and merged images **(Ad**,**h**–**Hd**,**h)**. In all Whrn mutant panels, Caspr **(Bb**,**d**,**f**,**h**; **Db**,**d**,**f**,**h**; **Eb**,**d**,**f**,**h**; **Fb**,**d**,**f**,**h**; **Gb**,**d**,**f**,**h**, **Ib**,**d**,**f**,**h**, green**)** fail to compact properly at the paranodes while juxtaparanodal marker K_v_1.2 **(Ba**,**Bd**; **Da**,**Dd**; **Ea**,**Ed**; **Fa**,**Fd**; **Ga**,**Gd**; **Ia**,**d**, red**)** appears diffuse and disorganized. Nodal NF186 **(Ac**-**Ic**, blue**)** appear unaffected by genotype or age. Cytoskeletal markers like Nfl-H **(De**-**Ge** vs. **Ce**, **Ie** vs. **He**, red**)** and 4.1B **(Dg**-**Gg** vs. **Cg**, **Ig** vs. **Hg**, blue**)** show more diffusion and irregularities in intensity, particularly older animals, in *Whrn* mutant nerves compared to wild-type nerves, suggesting Whrn contributes to paranodal cytoskeletal stability. Scale bars **(Ad**-**Id**, **Ah**-**Ih)** = 5 μm. Further quantification and percentages of phenotype-positive sciatic nerves is included for each age/genotype in a summary graph (bottom).

To expand on the characterization of Whrn loss with respect to myelinated domain organization, we examined a larger developmental window from postnatal ages 2 weeks to 1 year. Wild-type and *Whrn* knockout sciatic nerves revealed the following percentages of Caspr-stained phenotype-positive paranodes (Figure 2): 1.5% and 2.7% at 10 weeks, 0.9% and 1.8% at 20 weeks, 0.8% and 1.5% at 30 weeks, 0.5% and 1.4% at 40 weeks, and 0.7% and 1.5% at 1 year, respectively. Similar to the 4–8 week studies (Figure 2), we immunostained fibers with nodal, paranodal, and juxtaparanodal markers, as well as the axonal cytoskeletal protein markers 4.1B and heavy chain Neurofilament (Nfl-H) given Whrn’s known cytoskeletal scaffolding role. We observed no differences in nodal formation using NF186 (Figure 3Ac, Bc) in 2 week-old fibers. Abnormalities in paranodal formation and com-paction were observed in Caspr-stained *Whrn*^*−/−*^ fibers (Figure 3Bb, Bf) compared to wild-type (Figure 3Ab, Af). Overall K_v_1.2 juxtaparanodal signal appeared similar between 2 week-old wild-type (Figure 3Aa) and *Whrn*^*−/−*^ fibers (Figure 3Ba) with accumulation and enrichment of K_v_1.2 channels (Figure 3Bd) neighboring Caspr within the internodal region. No obvious difference in intensity or localization of Nfl-H (Figure 3Ae, Be) or 4.1B (Figure 3Ag, Bg) was found in 2 week-old sciatic nerve fibers between *Whrn* genotypes. With 10 week-old sciatic nerve fibers, we observed no differences in nodal domains using NF186 (Figure 3Cc, Dc). Like 8 week-old fibers (Figure 2F-H), we observed 10 week-old *Whrn*^*−/−*^ fibers with abnormal paranodal compaction (Figure 3Db, Df), when compared to wild-type fibers (Figure 3Cb, Cf). Juxtaparanodal domains stained with K_v_1.2 (Figure 3Da vs. 3Ca) appear more diffuse but similar in overall intensity in 10 week-old *Whrn*^*−/−*^ fibers when compared to wild-type. We observed slight enrichment of Nfl-H (Figure 3Ce vs. 3De) and 4.1B (Figure 3Cg vs. 3Dg) within the paranodal region of 10 week-old wild-type sciatic nerves compared to *Whrn* knockout mice, but the overall intensity of Nfl-H and 4.1B along the remaining axon appeared similar. Immunostaining of 20, 30, and 40 week-old wild-type sciatic nerve fibers were performed and domain organization was identical to 10 week-old wild-type fibers (Figure 3Ca-k). Looking at 20, 30, and 40 week-old *Whrn*^*−/−*^ peripheral nerve fibers, we observed no differences in nodal domains between *Whrn* wild-types (Figure 3Cc) or knockout mice using NF186 (Figure 3Ec-Gc). Similar to 10 week-old fibers (Figure 3Db, Df), we observed numerous 20, 30, and 40 week-old Whrn null fibers stained with Caspr (Figure 3Eb,d-Gb,d) with abnormal paranodal compaction when compared to wild-type fibers (Figure 3Cb,d). In 20, 30, and 40-week old *Whrn*^*−/−*^ fibers, juxtaparanodal domains stained with K_v_1.2 (Figure 3Ea-Ga) showed numerous breaks at sites of Caspr signal, appeared less symmetrical, and displayed similar overall signal when compared with wild-type (Figure 3Ca). Cytoskeletal marker Nfl-H staining (Figure 3Ee-Ge) was generally uniform within the axon, and 4.1B (Figure 3Eg-Gg) was present in all axonal domains except the node in 20, 30, and 40 week-old *Whrn*^*−/−*^ sciatic nerves. One-year-old sciatic nerves showed no difference in nodal organization (Figure 3Hc vs. 3Ic). One year-old *Whrn*^*−/−*^ sciatic nerves (Figure 3Ib,d) revealed abnormal paranodal compaction compared to wild-type fibers (Figure 3Hb,d). Notably, we observed paranodal compaction defects in older *Whrn*^*−/−*^ sciatic nerve fibers (40 week-old and 1 year-old) like blocks and bulges in Caspr signal as well as the spring-like phenotype observed in younger fibers (4–30 week-old). As seen previously, the juxtaparanodal domains of *Whrn*^*−/−*^ peripheral nerves stained with K_v_1.2 (Figure 3Ia) showed significant asymmetric enrichment and periodic breaks as well as overlap with Caspr signal when compared with one year-old wild-type fibers (Figure 3Ha). Also, patchy cytoskeletal-deficient staining was observed in older *Whrn*^*−/−*^ fibers (Figure 3Gh, 3Ih) near the paranode. Cytoskeletal marker Nfl-H staining was weakly enriched within the paranode of the wild-type axon (Figure 3Hh) and less uniform within *Whrn*^*−/−*^ fibers with paranodal compaction problems (Figure 3Ih). Likewise, 4.1B showed disrupted juxtaparanodal cytoskeletal staining in one year-old *Whrn*^*−/−*^ fibers (Figure 3Ig) which was not observed in the wild-type fibers (Figure 3Hg).

**Figure 4:**
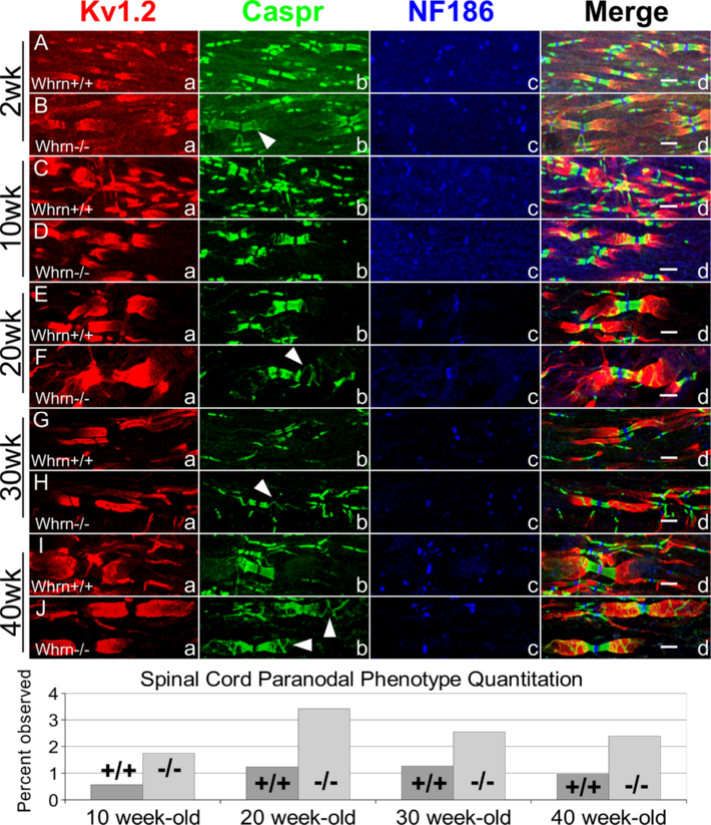
**Loss of Whirlin contributes to abnormal paranodal compaction throughout development in the spinal cord. A**–**J**. 2-week, 10, 20, 30, 40-week teased sciatic nerve fibers either wild-type **(Aa**–**Ad**, **Ca**–**Cd**, **Ea**–Ed, **Ga**–**Gd**, **Ia**–**Id)** or Whrn knockout **(Ba**–**Bd**, **Da**–**Dd**, **Fa**–**Fd**, **Ha**–**Hd**, **Ja**–**Jd)** immunostained against Caspr **(Ac**–**Jc**, green**)**, K_v_1.2 **(Aa**–**Ja**, red**)**, NF186 **(Ac**–**Jc**, blue**)**, and merged images **(Ad**–**Jd)**. Nodal NF186 **(Ac**–**Jc**, blue**)** appear unaffected by age or genotype. In Whrn mutant spinal cords, Caspr **(Bb**,**d**; **b**,**d**; **Fb**,**d**; **Hb**,**d**; **Jb**,**d**; green**)** does not compact properly at the paranodes compared to wild-type Caspr (**Ab**,**d**; **Cb**,**d**; **Eb**,**d**; **Gb**,**d**; **Ib**,**d**; green**)**. In addition, juxtaparanodal marker K_v_1.2 **(Ba**,**d**; **Da**,**d**; **Fa**,**d**; **Ha**,**d**; **Ja**,**d**; red**)** in *Whrn* mutant spinal cords shows similar signal intensity and occasional colocalization with Caspr-stained myelin loops. Scale bars **(Ad**–**Jd)** = 5 μm. Further quantification and percentages of phenotype-positive spinal cord fibers is included for each age/genotype in a summary graph (bottom).

We also sought to examine the effects of *Whrn* loss on axonal domain organization in the central nervous system (CNS). Utilizing white matter tracts in the spinal cord, we were able to identify by immunostaining subtle but consistent differences in paranodal compaction. As in the PNS (Figure 3), we stained longitudinal spinal cord sections with nodal, paranodal, and juxtaparanodal markers (Figure 4). Wild-type and *Whrn* knockout spinal cord fibers revealed the following percentages of Caspr-stained phenotype-positive paranodes (Figure 3): 0.6% and 1.7% at 10 weeks, 1.2% and 3.4% at 20 weeks, 1.3% and 2.5% at 30 weeks, and 1.0% and 2.4% at 40 weeks. No differences in nodal organization were observed (Figure 3.4Ac vs. 3.4Bc) in 2 week-old spinal cord sections. At 10, 20, 30, 40 week-old, nodal organization appeared similar between wild-type and *Whrn*^*−/−*^ spinal cord fibers using NF186 (Figure 4Cc vs. 4Dc, 4Ec vs. 4Fc, 4Gc vs. 4Hc, 4Ic vs. 4Jc). Immunostaining with paranodal Caspr revealed subtle and infrequent paranodal abnormalities in 2 week-old *Whrn* null spinal cord fibers (Figure 4Bb) compared to wild-type (Figure 4Ab). In contrast, obvious and regular paranodal compaction defects were observed in 10 (Figure 4Cb vs. 4Db), 20 (Figure 4Eb vs. 4Fb), 30 (Figure 4Gb vs. 4Hb), and 40 week-old (Figure 4Ib vs. 4Jb) *Whrn*^*−/−*^ spinal cords compared to their wild-type controls. Finally, juxtaparanodal K_v_1.2 immunostaining of 10 (Figure 4Ca vs. 4Da), 20 (Figure 4Ea vs. 4Fa), 30 (Figure 4Ga vs. 4Ha), and 40 week-old (Figure 4Ia vs. 4Ja) wild-type spinal cord myelinated axons showed similar overall expression as *Whrn* knockout axons, but consistent juxtaparanodal disorganization and colocalization with loosened Caspr-stained myelin loops was observed most often in *Whrn*^*−/−*^ fibers. In summary, there is significant evidence that loss of *Whrn* disrupts normal paranodal compaction in 10–40 week-old myelinated spinal cord axons with subsequent effects on juxtaparanodal organization. Taken together, both the peripheral and central nervous systems likely utilize the cytoskeletal properties of *Whrn* to help stabilize the cellular organization between myelinating glial cells and neurons around the paranodal region throughout development.

### Whirlin knockout mice have cerebellar Purkinje cells with bead-like, axonal swellings

**Figure 5:**
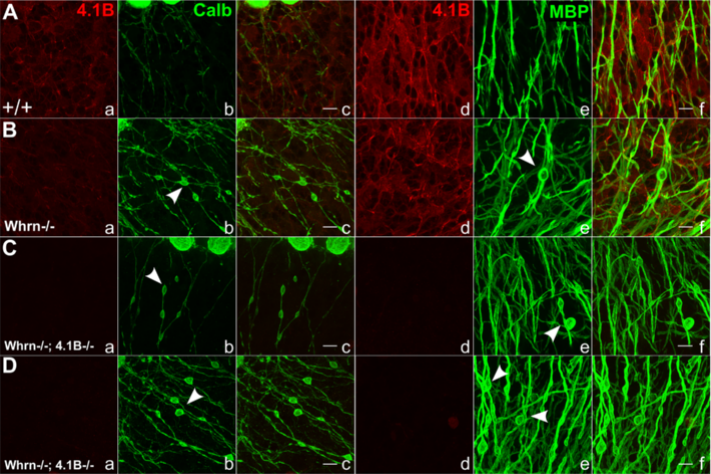
**Whirlin alone and combination 4.1B/Whirlin knockout mice have cerebellar Purkinje cells with bead-like, axonal swellings. A**–**D**. 6-week sagittal sections of mouse cerebellum in either wild-type **(Aa**–**Af)**, *Whrn* knockout **(Ba**–**Bf)**, or double Whrn knockout and 4.1B null **(Ca**–**Cf**, **Da**–**Df)** immunostained against Protein 4.1B band/4.1B **(Aa**–**Da**, **Ad**–**Dd**, red**)**, Calbindin **(Ab**–**Db**, green**)**, myelin basic protein/MBP **vAe**–**De**, green**)**, and merged images **(Ac**,**f**–**Dc**,**f)**. No striking difference was observed between wild-type and Whrn knockout slices in 4.1B staining **(Aa,d** vs. **Ba**,**d)** and only non-specific staining was present in 4.1B null animals **(Ca**–**Da**, **Cd**–**Dd)**. In Whrn mutant cerebellum sections **(Ba**–**Bf)**, Purkinje axonal swellings appear bead-like using both Calb **(Bb**,**c** vs. **Ab**,**c)** and MBP **(Be**,**f** vs. **Ae**,**f)** in comparison to a uniform, thin caliber axon in wild-type fibers **(Aa**–**f)**. Additionally, secondary ablation of 4.1B protein resulted in a greater number of swellings observed with Calb **(Cb**,**c**–**Db**,**c** vs. **Bb**,**c)** and MBP **(Ce**,**f**–**De**,**f** vs. **Be**,**f)** when compared with Whrn knockouts alone suggesting these cytoskeletal elements help prevent cytoskeletal disorganization in Purkinje cell axons. Scale bars **(Ac**–**Dc**, **Af**–**Df)** = 10 μm.

To determine the effects of Whrn loss on cerebellar Purkinje cell morphology, we immunostained cerebellar slices from 6 week-old wild-type, *Whrn* knockout, and double *Whrn* and *4.1B*[14] null animals (Figure 5). Given that *Caspr*[9] and *CGT*[28,29], two genes critical for formation of a proper paranode, display Purkinje axonal swellings and cytoskeletal disorganization, we were curious as to the effects of the combined loss of *Whrn* and *4.1B*. While no obvious differences in localization was observed using our Whrn antibody (data not shown) in any of these genotypes, we immunostained against 4.1B (Figure 5Aa,d–Da, d; red), Purkinje-specific Calbindin (Figure 5Ab–Db, green), and glial-specific myelin basic protein/MBP (Figure 5Ae–De, green). There was no difference in 4.1B staining intensity (Figure 5Aa,d vs. 5Ba,d) between wild-type and *Whrn* knockout slices. Upon examining *Whrn*^*−/−*^ cerebellum sections (Figure 5Ba–Bf), Purkinje axons appeared to contain bead-like, swellings along their extensions through the granular layer using both Calb (Figure 5Bb,c vs. 5Ab,c, white arrowheads) and MBP (Figure 5Be,f vs. 5Ae,f, white arrowheads) in comparison to a uniform caliber axon in wild-type fibers (Figure 5Ab,c,e,f). The secondary loss of 4.1B protein resulted in more swellings observed with Calb (Figure 5Cb,c– 5Db,c vs. 5Bb,c, white arrowheads) and MBP (Figure 5Ce,f– 5De,f vs. 5Be,f, white arrowheads) when compared with *Whrn* null animals alone. In summary, the cytoskeletal elements Whrn and 4.1B likely have an assistive role in preventing cytoskeletal accumulation and disorganization within cerebellar Purkinje cell axons in a similar phenotypic manner to Caspr null mice [9].

### Ultrastructural abnormalities in *Whrn* knockout sciatic nerve, spinal cord fibers, and cerebellar Purkinje axons

**Figure 6:**
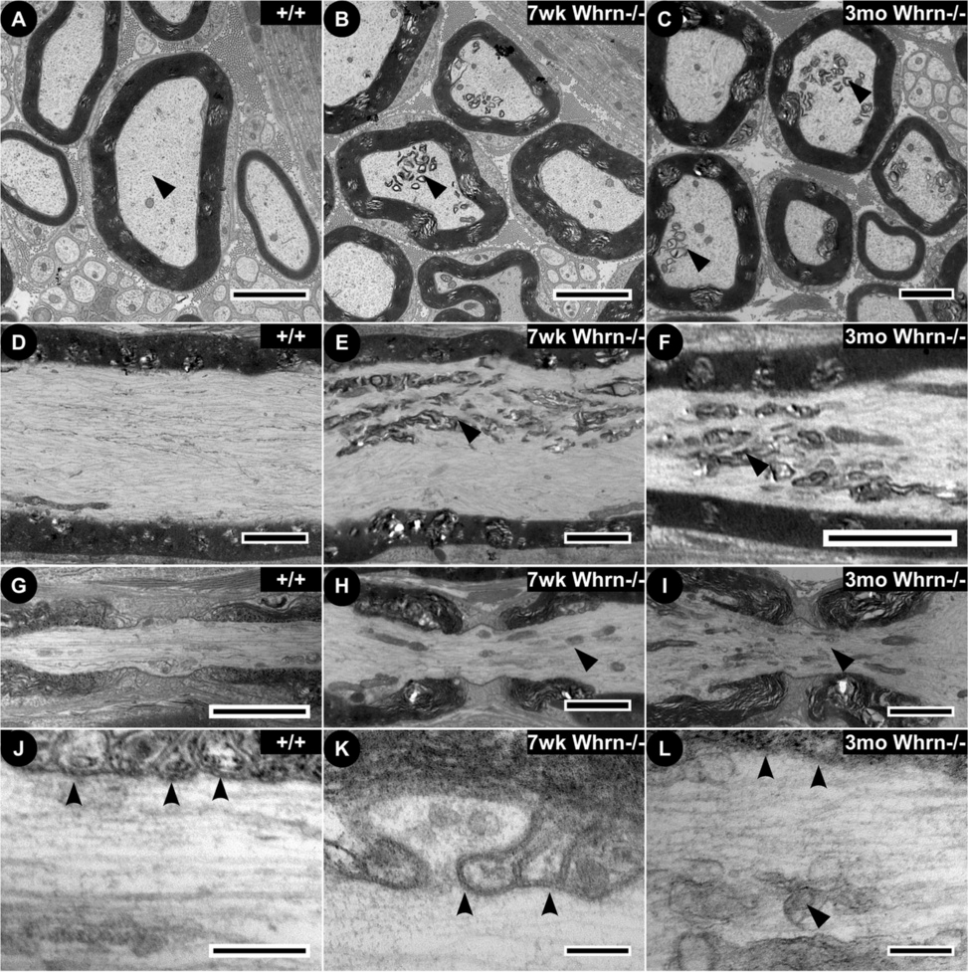
**Ultrastructural examination of *Whirlin *knockout sciatic nerves reveals organelle accumulation and cytoskeletal disruption.** Low magnification electron micrographs through the internodal regions of sciatic nerves in wild-type (7 week-old) and *Whrn* knockout mice (7 week-old, 3 month-old) in cross section **(A** vs. **B**, **C)** and longitudinal orientations **(D** vs. **E**, **F)**. Overall cellular organization between *Whrn* knockout and wild-type sciatic nerve fibers is conserved with tightly compacted myelin around each axon. Low magnification, longitudinal electron micrographs through the nodal and paranodal regions of sciatic nerves are presented for wild-type **(**7 week-old, **G)** and *Whrn* knockout mice (7 week-old, **H**; 3 month-old, **I**). At a higher magnification, the wild-type **(J)** paranodal loops have clearly defined characteristic transverse, electron-dense septa **(concave arrowheads)** and parallel arrays of cytoskeletal elements. In contrast, *Whrn* mutant paranodal septa **(K**, **L**, **concave arrowheads)** are less definitive and fuzzy with associated accumulation of organelles **(flat arrowheads)**, particularly mitochondria and transport vesicles. Scale bars: **A**–**I**, 2 μm,** J**–**L**, 400 nm.

**Figure 7:**
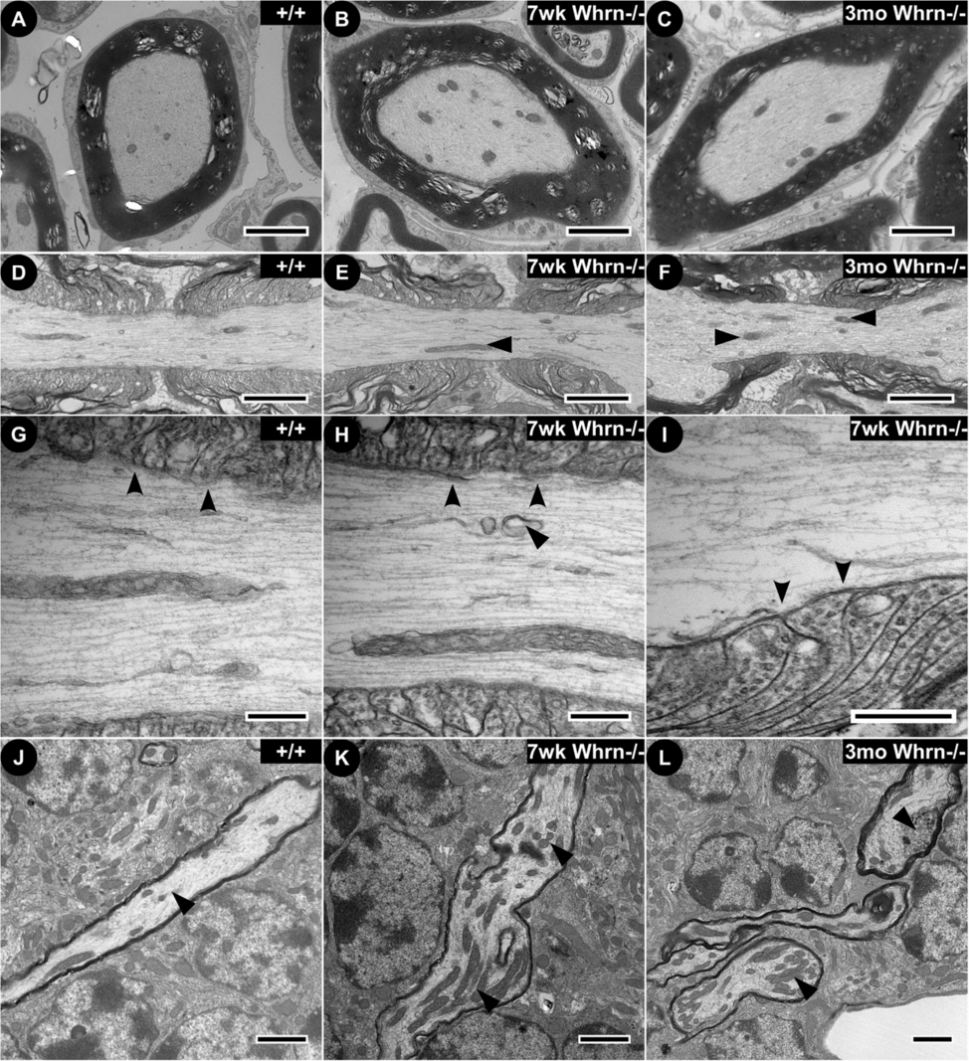
**Ultrastructural examination of *Whirlin *knockout central nervous system tissues reveals organelle accumulation in myelinated axons.** Electron micrographs of internodal cross sections **(A**–**C)** and nodal-paranodal longitudinal **(D**–**F)** regions in spinal cords from wild-type **(**7 week-old, **A**, **D)** and *Whrn* knockout mice **(**7 week-old, **B**, **E**; 3 month-old, **C**, **F)**. Overall cellular organization is conserved between wild-type and *Whrn* knockout and sciatic nerve fibers with tightly compacted myelin around each axon. Enrichment of mitochondria **(flat arrowheads)** and occasional myelin ruffling is observed in *Whrn* knockout mice compared to wild-type. At higher magnification, the wild-type **(G)** paranodal loops have characteristic electron-dense septa **(concave arrowheads)** and parallel arrays of cytoskeletal elements. In contrast, *Whrn* knockout CNS fibers accumulate organelles **(flat arrowheads)**, particularly mitochondria and transport vesicles, and have paranodal septa **(H**, **I**, **concave arrowheads)** that are less defined. Also, electron micrographs from mice show cerebellar Purkinje myelinated axons **(J**–**L)** running through the granular layer. This region reveals axonal swellings filled with densely-packed organelles **(flat arrowheads)**, particularly mitochondria and vesicles, in 7 week-old **(K)** and 3 month-old **(L)***Whrn* knockout animals compared to 7 week-old wild-type **(J)**. Scale bars: **A**–**F**, 2 μm, **G**–**I**, 400 nm, **J**–**L**: 1 μm.

To further understand Whrn’s role in myelinated axons, transmission electron microscopy was performed in order to examine the ultrastructural architecture in myelinated axons in the PNS (Figure 6) and CNS (Figure 7) of 7 week-old and 3 month-old wild-type and *Whrn*^*−/−*^ mice. Low-magnification, cross-section electron micrographs of wild-type (Figure 6A) and *Whrn* knockout (Figure 6B, C) myelinated sciatic nerve fibers showed the typical organization of tightly bound, electron-dense myelin wraps around the internodal region of the axonal membrane. Accumulation of mitochondria and lipid vesicles (Figure 6B, C vs. 6A and 6E, F vs. 6D, flat arrowheads) in the internodal regions was clearly observed in W*h*rn knockout animals compared to wild-type sciatic nerve fibers. Given the potential role of the mesaxon in the observed light microscope phenotype [15], we found no striking differences in the ultrastructural organization or arrangements of the inner mesaxon along the internodal region at 7 weeks or 3 months of age (data not shown). Consistent with our immunostaining data, no obvious differences in nodal organization were observed in either genotype (Figure 6G vs 6H,I). Higher magnification along the wild-type paranodal region (Figure 6J, concave arrowheads) revealed the hallmark electron-dense AGSJs formed between the glial paranodal loops and axolemma and accompanying parallel arrays of cytoskeletal elements in the axon. In contrast, the *Whrn* knockout paranodal region of 7 week-old (Figure 6K) and 3 month-old (Figure 6L) displayed poorly defined but present AGSJs (Figure 6K,L, concave arrowheads), less organized neurofilaments and microtubules, and consistent accumulation of mitochondria and lipid vesicles (Figure 6H, 6I, 6L, flat arrowheads) in the paranodal region. The ultrastructural phenotypes displayed at both 7 weeks and 3 months of age suggest that Whrn is important for the long-term maintenance and the overall structure of the myelinated axons.

In the central nervous system, low-magnification, electron micrographs of wild-type (Figure 7A) and *Whrn* knockout (Figure 7B, C) myelinated fibers showed slight differences in spinal cord cross-sections. Like the peripheral nerves, the overall organization between glial cell and neuron remained similar between wild-type and *Whrn*^*−/−*^ fibers. However, mitochondria were slightly more abundant in *Whrn* knockout fibers. No obvious ultrastructural differences were observed in the node of either genotype (Figure 7D-F), but greater accumulation of mitochondria (flat arrowheads) and lipid vesicles was observed in the paranodal and juxtaparanodal regions. Higher magnification along the paranodal region (Figure 7G-I, concave arrowheads) revealed the expected electron-dense AGSJs formed between myelin loops and the axolemma. Like in the PNS, the *Whrn* knockout paranodal region of 7 week-old (Figure 7H) and 3 month-old (Figure 7I) displayed poorly defined but present AGSJs (concave arrowheads) and accumulation of mitochondria and lipid vesicles (Figure 7H, flat arrowhead) in the paranodal region. Examining the cerebellum, we observed Purkinje axon swellings in *Whrn* knockout fibers (Figure 7K-L). Low-magnification electron micrographs of 7 week-old wild-type (Figure 7J) and *Whrn* knockout at 7 weeks (Figure 7K) and 3 months (Figure 7L) of age showed striking differences in Purkinje axon myelinated fibers in the cerebellum granular layer. This region shows axonal swellings filled with densely-packed organelles, particularly mitochondria and vesicles (Figure 7K-L, flat arrowheads). Taken together, the ultrastructural analyses of *Whrn* knockout mice demonstrate that Whrn is critical for the stability of paranodal organization, proper axonal cytoskeletal arrangements, and prevention of sub-cellular organelle accumulation in myelinated axons.

## Conclusions

Cellular and molecular interactions between neurons and glia establish and stratify the numerous tasks of the nervous system. In particular, linkage of cellular membranes with the underlying cytoskeleton via cytoskeletal linker proteins helps maintain the specialized cellular arrangements necessary to glial and neuronal function. The potential of Whrn to link plasma membrane proteins with multiple cytoskeletal protein partners has been well established [16,17,19-24], yet a precise role for Whrn in the central or peripheral nervous system or even more specifically in myelinated neurons has not been examined. In myelinated axons, axonal membrane proteins like Caspr and Caspr2 help stabilize domain organization through linkage of 4.1B to the underlying cytoskeleton. This loss of organization at both the light and electron microscopy level is readily apparent in mutant mice lacking Caspr [7], Caspr2 [12], and 4.1B [14]. Here we report that *Whrn* knockout animals reveal defective cytoskeletal organization and accumulation of organelles in the myelinated fibers. Our phenotypic analyses of *Whrn* knockout mice demonstrate that loss of *Whrn* disrupts proper paranodal compaction and long-term stability of the myelinated axons throughout development.

### Whrn alternative splicing and phenotypes

To understand the function of Whrn in the nervous system, one must be considerate of *Whrn* mRNA splice variants and rule out confounding mouse genetic strain differences. Currently, two mutant mouse strains exist for *Whirlin*: the *whirler* (*Whrn*^*wi/wi*^) mouse has a spontaneous genomic deletion of exons 6–9 while the *Whrn* (*Whrn*^*−/−*^) knockout mouse [17] has a targeted exon 1 deletion. We examined the localization of several myelinated axon markers (Caspr, 4.1B, K_v_1.2, and NF186) in Whrn wild-type mice from each background strain and observed no disruptions in the localization of these proteins or the morphology of the paranodes (data not shown). Our initial studies comparing *Whrn*^*−/−*^ to *Whrn*^*wi/wi*^ mouse strains suggested myelinated domain organization, early paranodal disorganization, and nerve conduction (~30 m/s) was indistinguishable between each mutant mouse line. Given the novelty of the phenotype, we additionally substantiated no mouse strain effects after back-crossing to isogenic *C57BL6* mice for two generations. Given the consistency of the mutant phenotype regardless of background mouse genetics, we were assured the phenotype was attributed to loss of *Whrn* function and not alternative *Whrn* splice variants or mouse genetic background variation.

### Scaffolding by Whrn and previously established protein networks may underlie paranodal compaction and stabilization

Whrn has several established roles derived from its complex and numerous interactions with other protein partners within the ear and eye. This complexity comes from identifying and comparing the human and mouse *Whrn* mutants and splice variants. The significant bulk of Whrn research has been performed in the ear and eye since human *WHRN* mutations contribute to a subset of Usher syndromes. In the eye, full-length Whrn colocalizes with the transmembrane proteins Usherin and VLGR1 at the periciliary membrane complex in photoreceptors [17]. Studies demonstrate the two N-terminal PDZ domains of the full-length Whrn isoform are responsible for this interaction. In the ear, the shorter Whrn isoform has a more significant role since mutation and/or loss of Whrn’s C-terminus correlates to poorer hearing deficits compared to Whrn’s N-terminus. The Whrn short isoform interacts through its Proline-rich domain and last PDZ domain with Myosin XVa and Mpp1/p55. Additional reports demonstrate Whrn protein expression in the mouse cerebrum, cerebellum, and brainstem [25], rat cerebellum [27], and in the *Drosophila* central nervous system [26]. Taken together, Whrn has a well-documented history of shared protein-protein interaction across multiple model systems and cells.

We propose that Whrn as a cytoskeletal scaffold crosslinks a subcellular axonal meshwork to stabilize the paranode, juxtaparanode, or both, and that in the absence of Whrn, subcellular compaction of the paranode and organization of underlying microtubules, neurofilaments, cause cytoskeletal disorganization leading to an accumulation of mitochondria, and lipid vesicles along myelinated axons. Here we propose the potential sites of interaction for Whrn within these regions given its established protein-protein interactions *in vivo*. Within the paranode, the intracellular c-terminus of Caspr contains a SH3 domain, a potential site of interaction with Whrn’s Proline-rich domain. The paranode and juxtaparanode are also enriched for 4.1B, a known protein partner in the ear stereocilia for Mpp1/p55 which interacts with Whrn [22]. Caspr2 also contains a PDZ-binding motif which could potentially interact with one of Whrn’s PDZ domains [12]. Finally, the c-terminus of Whrn has the potential for self-oligomerization [20], allowing for even more complex networks of protein-protein interaction within the myelinated axon.

Domain organization in myelinated axons is a complicated developmental process, culminating from intrinsic and extrinsic cellular factors. Here we demonstrate that Whrn expression is important for proper axonal domain organization and expand the role of Whrn outside the ear and eye. The phenotypes observed in the myelinated axons highlight that the paranodal-juxtaparanodal interface represents a substantial region for insight into paranodal stabilization and potential interaction with the axonal cytoskeleton. In light of recent evidence of 4.1G’s role at the internode and mesaxon, this paranodal-juxtaparanodal interface may represent an important subdomain in the study of myelinated fibers. Whrn’s protein domains have the potential to stabilize the paranodal myelin loops and associated cytoskeleton through direct or indirect interactions with Caspr, 4.1B, or other unidentified cytoskeletal proteins. These observations are correlated using several techniques including biochemistry, light and electron microscopy. Our observed paranodal phenotypes are consistent throughout development (2 wk-1 yr) and similar between central and peripheral nervous systems. One final important consideration about cytoskeletal linker proteins, both *Whrn* and *4.1B* null mice have no statistical difference in conduction velocities in sciatic nerves compared to wild-type mice, despite having clear paranodal instability in Caspr-stained myelinated fibers. Such data suggest cytoskeletal linker proteins may be functionally redundant with respect to myelinated domain organization and may require secondary or tertiary genetic ablations to achieve any measurable electrophysiological effects. To this point, we observed the increase in Purkinje axonal swellings in the double *Whrn*; *4.1B* null mouse cerebellum compared to the single *Whrn* knockout or wild-type mouse cerebellum. In conclusion, our observations indicate Whrn acts as a cytoskeletal scaffolding protein that is essential for proper paranodal compaction and stabilization of the axonal cytoskeleton for long-term health of myelinated axons.

## Methods

### Animals

*Whrn* exon 1 homozygous mutants used were obtained from Dr. Jun Yang’s lab (University of Utah, Salt Lake City, Utah, 84132). The mice were backcrossed to *C57BL6* mice (JAX Laboratories #000664, Bar Harbor, Maine) for two generations and maintained as heterozygous *Whrn*^*+/−*^ breeding stocks. All animal experiments were performed according to Institutional Animal Care and Use Committee approved guidelines for ethical treatment of laboratory animals at the University of North Carolina at Chapel Hill and the University of Texas Health Science Center at San Antonio.

### Genotyping

Genomic DNA from mouse tails and/or toe snips was extracted using a kit according to manufacturer’s instructions (Sigma-Aldrich Extract-N-Amp™ Tissue PCR Kit (XNAT2)). Cycling conditions used were: 95°C for 5 min, 35 cycles of (95°C for 30 sec, 68°C for 1.5 min), and 68°C for 3 min. Primer sequences for PCR were obtained from Dr. Jun Yang’s lab as follows: common WhrnRP (Pdzg5r) CAGGGAAGTTGAGGCACACGG, wild-type Whrn+FP (pdzg1) GGGTGAGTGAATGCCAGCCAG, and knockout Whrn-FP (PNT3A) GAGATCAGCAGCCTCTGTTCCAC. The Whrn+ product size is 894 bp; the Whrn- product size is 700 bp.

### Generation of Whrn antibody

We generated rabbit, guinea pig, and rat polyclonal anti-Whrn antibodies similar to previous literature [23]. A full length *Whrn* mouse cDNA construct in pcDNA3.1 was obtained from Dr. Jun Yang’s lab. Regions encoding amino-acid residues 220–326 and 699–804 of mouse *Whirlin* (Genbank: NP_001008791.1) were subcloned individually into pGEX4T1 and expressed in Escherichia coli (BL21; Stratagene, La Jolla, CA). Fusion proteins were isolated by incubating with Glutathione Sepharose 4 Fast Flow (GE Healthcare, Sweden). Each fusion protein was used to immunize a rabbit, guinea pig, or rat (Cocalico Biologicals Inc, Reamstown, PA). cDNAs encoding amino acids 220–326 or 699–804 of mouse Whrn were also introduced into pMAL-c2x (New England Biolabs, Beverely, MA), transformed into E. coli (DE3 BL21; Stratagene) and induced to express the corresponding maltose binding (MBP) fusion protein. The expressed MBP-fusion proteins were purified using amylose resin (New England Biolabs, Beverely, MA) and then linked to a NHS-activated Sepharose 4 Fast Flow (GE Healthcare, Sweden). Only antisera from the immunized rabbit #349 (RbWhrn349) was affinity purified using the corresponding MBP–Whrn fusion protein.

### RNA preparation and RT-PCR

Sciatic nerves, dorsal root ganglia, and spinal cord sections were removed from p21 *Whrn* mutant and wild-type mice. Tissue was stored and processed in RNAlater Stabilization Reagent (QIAGEN). Total RNA was isolated using QIAshredder columns (QIAGEN) and RNeasy Mini Kit (QIAGEN). RNA concentration was estimated and approx. 5 ng RNA was used for reverse transcription followed by PCR amplification using the MyTaq One-Step RT-PCR kit (Bioline). RT-PCR analysis was performed on three separate sets of animals. Cycling conditions used were: 45°C for 20 min, 95°C for 1 min, 35 cycles of (95°C for 10 sec, 62°C for 10 sec, 72°C for 30 sec), and 72°C for 5 min. Primers used for PCR were as follows: *Actin* (Ex2) FP: GCTCCGGCATGTGCAA, *Actin* (Ex4) RP: AGGATCTTCATGAGGTAGT. *Whrn* (Ex1) FP: ACCAGATTCTGCGCGTCAAC, *Whrn* (SalI-Ex4) RP: tccgGTCGACcacctccagaatctggtctc; *Whrn* (EcoRI-Ex9) FP: CCCAgaattcGGGGCCTGCCTTCCACC, *Whrn* (SalI-Ex10) RP: cgggGTCGACgttggcaccctccgcgg.

### Other antibodies and immunostaining reagents

The following antisera were previously described: guinea pig and rabbit anti-Caspr [7,10,30], guinea pig anti-NF186 and guinea pig anti-pan Neurofascin [10,30], guinea pig anti-4.1B antibodies [14], and mouse anti-Calbindin [31]. Other primary antibodies used include the following: mouse anti- K_v_1.2 (University of California Davis/NIH NeuroMab Facility; K14/16), mouse anti-CASK (University of California Davis/NIH NeuroMab Facility; K56A/50), mouse anti-Ankyrin G (University of California Davis/NIH NeuroMab Facility; N106/36), mouse anti-Neurofilament H (Chemicon, MAB1623), rabbit anti-alpha-Tubulin (Cell Signaling #2144) and anti-Myelin Basic Protein (Abcam, SMI-94). Secondary antibodies used for immunofluorescence were Alexa Fluor-488, -568, and −647 conjugated (Invitrogen). HRP-conjugated secondary antibodies were purchased from Jackson ImmunoResearch.

### Immunostaining

Briefly, sciatic nerves were removed from anesthetized littermate wild-type and *Whrn* mutants of either sex and fixed in 4% paraformaldehyde in PBS for 15–30 min. The nerves were washed with PBS three times (10 min each) and stored at 4°C until teased. The nerves were teased into individual fibers in PBS, mounted on glass slides, and dried overnight at room temperature. Fibers were either immediately used for immunostaining or stored at −80°C until needed. Teased nerve slides were submerged in acetone (methanol instead for anti-MBP staining) at −20°C for 20 min then washed with PBS, followed by immunostaining [4]. For spinal cord sections and cerebellar sections, wild-type and mutants were deeply anesthetized and intra-cardially perfused with PBS followed by ice-cold 4% paraformaldehyde in PBS. The spinal cord or cerebellum was dissected out and post-fixed in 4% paraformaldehyde overnight at 4°C. The tissues were rinsed with PBS and sectioned to 30 um using a Vibratome (Leica). The sections were then immediately immunostained as previously described [9,10]. Primary antibodies for immunostaining were used at the following concentrations overnight at 4°C: RbCaspr @1:500, GPNF186 @1:400, MsIgG2b- K_v_1.2 @1:200, GPNFCt @1:400, GP-beta-IVspectrin @1:1000, GP4.1B @1:10000, and MsIgG-Nfl-H @1:1000, MsIgG1-Calb @1:1000, and MsIgG1-MBP @1:200.

### Immunoblotting

Sciatic nerves and dorsal root ganglia from littermate wild-type and mutants of either sex were excised and processed using a glass homogenizer in ice-cold lysis buffer (50 mM Tris–HCl, pH 7.5, 150 mM NaCl, 10 mM EDTA, 1% Triton X-100, 1% SDS, and a protease mixture tablet). The lysate was incubated for 30 min on ice and then centrifuged at 16,000×g for 20 min at 4°C. The sciatic nerve or dorsal root ganglia supernatant was saved for further processing. Spinal cords from littermate wild-type and mutants of either sex were excised and either directly processed or frozen at −80°C. Spinal cords were homogenized using a glass mortar and pestle on ice with lysis buffer (50 mM Tris–HCl, pH 7.5, 150 mM NaCl, 10 mM EDTA, 1% Triton X-100, and a protease inhibitor mixture tablet) and incubated for 30 min on ice with occasional trituration. The homogenate was centrifuged at 1000xg for 10 min at 4°C. The supernatant was collected and subjected to an additional centrifugation at 100,000 × g for 30 min at 4°C. The resulting second supernatant was collected and saved for further processing. Protein concentrations of final lysates were determined using the Lowry assay (BC assay; Bio-Rad). Lysates were resolved by SDS-PAGE and transferred onto nitrocellulose membranes, followed by immunoblotting procedures described previously [4]. Primary antibodies for immunoblotting were used at the following concentrations for 1 hr at room temperature: Affinity-purified RbWhrn349 @ 1:1000 (overnight at 4°C), GPCaspr @ 1:2000, GPNFCt @ 1:2000, Rb4.1B @ 1:50000, MsIgG1-CASK @ 1:50000, RbCaspr2 @ 1:50000, RbTubulin @ 1:2000 (overnight at 4°C).

### Electrophysiology

The conduction velocity measurements of sciatic nerves were carried out on three separate wild-type (+/+) and *Whrn*^*−/−*^ mice as described previously [10,30].

### Image analysis and software

Confocal images were captured with a Zeiss LSM510 microscope. Scanning parameters were optimized for wild-type tissues and maintained for scanning the mutant tissues. Immunofluorescence images for sciatic nerves and spinal cords are composite projections from Z stacks of three to six sections (0.6um scan step) or stacks of ten to twenty sections (0.6um scan step) for cerebellar slices. Software used for assembling figures included Zeiss LSM Image Browser (v4.2), ImageJ (v1.47d), GIMP (v2.82), and OpenOffice (v3.4.1).

### Quantification of phenotype and statistics

For the initial quantification, we utilized a blinded counting strategy to best estimate the spring-like phenotype. Teased sciatic nerves were prepared from wild-type or *Whrn*^*−/−*^ mice. One individual prepared all teased slides and randomly assigned a number to each slide. Once completed, the individual compiled a table of genotypes matched to assigned numbers. Blinded to that table, a second individual immunostained the numbered slides and counted wild-type and *Whrn*^*−/−*^ Caspr-stained paranodes. Immunostained paranodes were counted under a fluorescent microscope at 40× magnification. Any paranode with 3 or more spring-like, loops were considered phenotype-positive. Data was compiled as the percentage of phenotype-positive paranodes out of total paranodes. Phenotype percentages per slide were matched to genotype and then a final average phenotype percent for wild-type or *Whrn*^*−/−*^ fibers was calculated as well the standard error of the mean (SEM). A standard t-test was used to calculate the statistical significance (p-value) between the percent for 7 week-old wild-type or *Whrn*^*−/−*^ fibers. Similar phenotype quantitation was applied to 10, 20, 30, and 40 wk old sciatic nerve and spinal cords. All measured data was tabulated in Microsoft Excel. A p-value of 0.05 was considered to indicate a significant difference between groups.

For secondary quantitation of paranodal parameters in 4–8 week-old animals, Caspr-stained paranodes were imaged using a confocal LSM510 microscope and accompanying Zeiss Image software. A composite projection of Z-stacks was performed for each image. Large-caliber sciatic nerve images (~10um total diameter glial-edge to glial edge) were analyzed given the initial observation of more phenotype-positive paranodes in large vs. small caliber axons. Twenty wild-type and twenty *Whrn*^*−/−*^ paranode images were selected for each time point (60 total images). Using Zeiss software tools, each paranode was measured in microns for nodal gap (distance between nodal-paranodal boundaries in white), paranodal diameter (distance across axon caliber in blue), paranodal width (distance from nodal-paranodal boundary to paranodal-juxtaparanodal boundary in red), and phenotype percentage (a paranode was considered phenotype-positive if the purple line from the paranodal-juxtaparanodal boundary crossed a Caspr AGSJ line three times (i.e. 1.5 circular myelin wrapping loops) and phenotype-negative if less than three times). All measured data was tabulated in Microsoft Excel. The average and standard deviation of each age and genotype was graphed for nodal gap, paranodal diameter, and paranodal width. Phenotype percentage was reported as a percent of phenotype-positive paranodes out of twenty total counted for that age and genotype.

### Transmission electron microscopy

Animal tissues were fixed using 4% Formaldehyde/1% Glutaraldehyde (4CF1G) via intra-cardiac perfusion for 30 minutes. Tissues were dissected out and placed in 4CF1G to post-fix overnight at 4°C. Tissues were then processed by UTHSCSA Electron Microscopy core. Core processing steps as follows: (1) buffer rinse in 0.1 M phosphate buffer for 5 minutes to overnight, (2) post-fixation in 1% Zetterqvist’s buffered Osmium Tetroxide for 30 minutes, (3) buffer rinse in Zetterqvist’s buffer for 3 minutes, (4) en bloc staining in 2% aqueous uranly acetate for 20 minutes, (5) dehydration in 70% alcohol for 10 minutes, then 95% alcohol for 10 minutes, then 100% alcohol twice for 10 minutes, then propylene oxide twice for 10 minutes, (5) resin infiltration in 1:1 propylene oxide:resin for 30 minutes then 100% resin for 30 minutes under 25 psi vacuum. Once embedded, tissue was sliced in 90 nm sections and placed on copper grids. Grids were stained with uranyl acetate for 30 seconds in the microwave and then with Reynold’s lead for 20 seconds. Samples were imaged at 80 kV on a JEOL 1230 electron microscope using AMT (advanced microscopy techniques) software.

## Abbreviations

AGSJ: Axo-glial septate junction; Whrn: Whirlin; Caspr: Contactin-associated protein; 4.1B: Protein 4.1B (brain) band.

## Competing interests

The authors declared that they have no competing interests.

## Authors’ contributions

JG performed genotyping and phenotype quantitation, immunostaining and confocal imaging; JY provided *Whrn* exon 1 knockout mouse strain; MG and BK provided *whirler* mouse strain and various Whrn-related reagents; JG and MB participated in the design of the study, assembled figures, and drafting of the manuscript. All authors approved the final manuscript.

## References

[B1] Bhat MA Molecular organization of axo-glial junctions Curr Opin Neurobiol 2003 13 5 552 559 10.1016/j.conb.2003.09.004 14630217

[B2] Salzer JL Polarized domains of myelinated axons Neuron 2003 40 2 297 318 10.1016/S0896-6273(03)00628-7 14556710

[B3] Thaxton C Bhat MA Myelination and regional domain differentiation of the axon Results Probl Cell Differ 2009 48 1 28 10.1007/400_2009_1 19343313 PMC2824168

[B4] Thaxton C Pillai AM Pribisko AL Dupree JL Bhat MA Nodes of Ranvier act as barriers to restrict invasion of flanking paranodal domains in myelinated axons Neuron 2011 69 2 244 257 10.1016/j.neuron.2010.12.016 21262464 PMC3035172

[B5] Rosenbluth J Role of glial cells in the differentiation and function of myelinated axons Int J Dev Neurosci 1988 6 1 3 24 10.1016/0736-5748(88)90025-1 3213568

[B6] Boyle ME Berglund EO Murai KK Weber L Peles E Ranscht B Contactin orchestrates assembly of the septate-like junctions at the paranode in myelinated peripheral nerve Neuron 2001 30 2 385 397 10.1016/S0896-6273(01)00296-3 11395001

[B7] Bhat MA Rios JC Lu Y Garcia-Fresco GP Ching W St Martin M Li J Einheber S Chesler M Rosenbluth J Axon-glia interactions and the domain organization of myelinated axons requires neurexin IV/Caspr/Paranodin Neuron 2001 30 2 369 383 10.1016/S0896-6273(01)00294-X 11395000

[B8] Tait S Gunn-Moore F Collinson JM Huang J Lubetzki C Pedraza L Sherman DL Colman DR Brophy PJ An oligodendrocyte cell adhesion molecule at the site of assembly of the paranodal axo-glial junction J Cell Biol 2000 150 3 657 666 10.1083/jcb.150.3.657 10931875 PMC2175192

[B9] Garcia-Fresco GP Sousa AD Pillai AM Moy SS Crawley JN Tessarollo L Dupree JL Bhat MA Disruption of axo-glial junctions causes cytoskeletal disorganization and degeneration of Purkinje neuron axons Proc Natl Acad Sci U S A 2006 103 13 5137 5142 10.1073/pnas.0601082103 16551741 PMC1405910

[B10] Pillai AM Thaxton C Pribisko AL Cheng JG Dupree JL Bhat MA Spatiotemporal ablation of myelinating glia-specific neurofascin (Nfasc NF155) in mice reveals gradual loss of paranodal axoglial junctions and concomitant disorganization of axonal domains J Neurosci Res 2009 87 8 1773 1793 10.1002/jnr.22015 19185024 PMC2837286

[B11] Zonta B Tait S Melrose S Anderson H Harroch S Higginson J Sherman DL Brophy PJ Glial and neuronal isoforms of Neurofascin have distinct roles in the assembly of nodes of Ranvier in the central nervous system J Cell Biol 2008 181 7 1169 1177 10.1083/jcb.200712154 18573915 PMC2442198

[B12] Poliak S Salomon D Elhanany H Sabanay H Kiernan B Pevny L Stewart CL Xu X Chiu SY Shrager P Juxtaparanodal clustering of Shaker-like K+ channels in myelinated axons depends on Caspr2 and TAG-1 J Cell Biol 2003 162 6 1149 1160 10.1083/jcb.200305018 12963709 PMC2172860

[B13] Horresh I Bar V Kissil JL Peles E Organization of myelinated axons by Caspr and Caspr2 requires the cytoskeletal adapter protein 4.1B J Neurosci: the official journal of the Society for Neuroscience 2010 30 7 2480 2489 10.1523/JNEUROSCI.5225-09.2010 PMC2836844 20164332

[B14] Buttermore ED Dupree JL Cheng J An X Tessarollo L Bhat MA The cytoskeletal adaptor protein band 4.1B is required for the maintenance of paranodal axoglial septate junctions in myelinated axons J Neurosci: the official journal of the Society for Neuroscience 2011 31 22 8013 8024 10.1523/JNEUROSCI.1015-11.2011 PMC3121702 21632923

[B15] Ivanovic A Horresh I Golan N Spiegel I Sabanay H Frechter S Ohno S Terada N Mobius W Rosenbluth J The cytoskeletal adapter protein 4.1G organizes the internodes in peripheral myelinated nerves J Cell Biol 2012 196 3 337 344 10.1083/jcb.201111127 22291039 PMC3275379

[B16] Mburu P Mustapha M Varela A Weil D El-Amraoui A Holme RH Rump A Hardisty RE Blanchard S Coimbra RS Defects in whirlin, a PDZ domain molecule involved in stereocilia elongation, cause deafness in the whirler mouse and families with DFNB31 Nature genetics 2003 34 4 421 428 10.1038/ng1208 12833159

[B17] Yang J Liu X Zhao Y Adamian M Pawlyk B Sun X McMillan DR Liberman MC Li T Ablation of whirlin long isoform disrupts the USH2 protein complex and causes vision and hearing loss PLoS genetics 2010 6 5 e1000955 10.1371/journal.pgen.1000955 20502675 PMC2873905

[B18] Friedman TB Schultz JM Ahmed ZM Tsilou ET Brewer CC Usher syndrome: hearing loss with vision loss Adv Oto-Rhino-Laryng 2011 70 56 65 10.1159/000322473 21358186

[B19] van Wijk E van der Zwaag B Peters T Zimmermann U Te Brinke H Kersten FF Marker T Aller E Hoefsloot LH Cremers CW The DFNB31 gene product whirlin connects to the Usher protein network in the cochlea and retina by direct association with USH2A and VLGR1 Hum Mol Genet 2006 15 5 751 765 10.1093/hmg/ddi490 16434480

[B20] Delprat B Michel V Goodyear R Yamasaki Y Michalski N El-Amraoui A Perfettini I Legrain P Richardson G Hardelin JP Myosin XVa and whirlin, two deafness gene products required for hair bundle growth, are located at the stereocilia tips and interact directly Hum Mol Genet 2005 14 3 401 410 15590698 10.1093/hmg/ddi036

[B21] Okumura K Mochizuki E Yokohama M Yamakawa H Shitara H Mburu P Yonekawa H Brown SD Kikkawa Y Protein 4.1 expression in the developing hair cells of the mouse inner ear Brain Res 2010 1307 53 62 19853587 10.1016/j.brainres.2009.10.039

[B22] Mburu P Kikkawa Y Townsend S Romero R Yonekawa H Brown SD Whirlin complexes with p55 at the stereocilia tip during hair cell development Proc Natl Acad Sci U S A 2006 103 29 10973 10978 10.1073/pnas.0600923103 16829577 PMC1544159

[B23] Belyantseva IA Boger ET Naz S Frolenkov GI Sellers JR Ahmed ZM Griffith AJ Friedman TB Myosin-XVa is required for tip localization of whirlin and differential elongation of hair-cell stereocilia Nat Cell Biol 2005 7 2 148 156 10.1038/ncb1219 15654330

[B24] Manor U Disanza A Grati M Andrade L Lin H Di Fiore PP Scita G Kachar B Regulation of stereocilia length by myosin XVa and whirlin depends on the actin-regulatory protein Eps8 Curr Biol : CB 2011 21 2 167 172 10.1016/j.cub.2010.12.046 21236676 PMC3040242

[B25] Wang L Zou J Shen Z Song E Yang J Whirlin interacts with espin and modulates its actin-regulatory function: an insight into the mechanism of Usher syndrome type II Hum Mol Genet 2012 21 3 692 710 10.1093/hmg/ddr503 22048959 PMC3259019

[B26] Jepson JE Shahidullah M Lamaze A Peterson D Pan H Koh K Dyschronic, a Drosophila homolog of a deaf-blindness gene, regulates circadian output and Slowpoke channels PLoS genetics 2012 8 4 e1002671 10.1371/journal.pgen.1002671 22532808 PMC3330124

[B27] Yap CC Liang F Yamazaki Y Muto Y Kishida H Hayashida T Hashikawa T Yano R CIP98, a novel PDZ domain protein, is expressed in the central nervous system and interacts with calmodulin-dependent serine kinase JNeurochem 2003 85 1 123 134 10.1046/j.1471-4159.2003.01647.x 12641734

[B28] Dupree JL Girault JA Popko B Axo-glial interactions regulate the localization of axonal paranodal proteins J Cell Biol 1999 147 6 1145 1152 10.1083/jcb.147.6.1145 10601330 PMC2168103

[B29] Marcus J Dupree JL Popko B Myelin-associated glycoprotein and myelin galactolipids stabilize developing axo-glial interactions J Cell Biol 2002 156 3 567 577 10.1083/jcb.200111047 11827985 PMC2173329

[B30] Thaxton C Pillai AM Pribisko AL Labasque M Dupree JL Faivre-Sarrailh C Bhat MA In vivo deletion of immunoglobulin domains 5 and 6 in neurofascin (Nfasc) reveals domain-specific requirements in myelinated axons J Neurosci: the official journal of the Society for Neuroscience 2010 30 14 4868 4876 10.1523/JNEUROSCI.5951-09.2010 PMC2856701 20371806

[B31] Buttermore ED Piochon C Wallace ML Philpot BD Hansel C Bhat MA Pinceau organization in the cerebellum requires distinct functions of neurofascin in Purkinje and basket neurons during postnatal development J Neurosci: the official journal of the Society for Neuroscience 2012 32 14 4724 4742 10.1523/JNEUROSCI.5602-11.2012 PMC3337041 22492029

